# General Blending Models for Data From Mixture Experiments

**DOI:** 10.1080/00401706.2014.947003

**Published:** 2015-11-16

**Authors:** L. Brown, A. N. Donev, A. C. Bissett

**Affiliations:** ^a^ University of Manchester, Oxford Road, Manchester, M13 9PL, UK(Liam.Brown@manchester.ac.uk; A.N.Donev@manchester.ac.uk); ^b^ Federal-Mogul Friction Products Ltd, High Peak, Derbyshire, SK23 0JP, UK(Alastair.Bissett@federalmogul.com)

**Keywords:** Becker’s models, Model selection, Nonlinear models, Scheffé polynomials.

## Abstract

We propose a new class of models providing a powerful unification and extension of existing statistical methodology for analysis of data obtained in mixture experiments. These models, which integrate models proposed by Scheffé and Becker, extend considerably the range of mixture component effects that may be described. They become complex when the studied phenomenon requires it, but remain simple whenever possible. This article has supplementary material online.

## 1. INTRODUCTION

We introduce a new class of statistical models for mixture experiments. In such experiments, the response depends on the proportions of the mixture components, but not on the amount of the mixture. For example, the strength of an alloy depends on the proportions of the metals of which it is comprised. Similarly, many features of friction materials (such as their friction coefficients and compressibilities) depend on the proportions of the chemicals from which they are made.

The common practice for analyzing mixture experiments has evolved from the work of Scheffé (1958, 1963). Scheffé suggested the canonical polynomial models, which have provided the recourse for the majority of practitioners since, although alternatives have been proposed.

Quenouillé (1953, 1959, 1963) demonstrated that the Scheffé polynomials are incapable of describing common linear blending, a structure which he considers intuitively sensible. A component blends linearly when the effect of increasing its presence in the mixture, while keeping all other components in fixed relative proportions to each other, may be described by a linear relationship. For example, this is the effect of a dilutent. In response, Becker (1968) suggested alternative models whose terms assume such effects. Where necessary, the veracity of this assumption may be judged by any practitioner who applies Becker’s models, but this is equally true of the contrasting assumption in the Scheffé polynomials. Becker (1978) proposed related developments by introducing terms capable of describing a far broader range of effects than previously considered.

Prior to the work of Scheffé, ordinary polynomial models in mathematically independent variables (MIV) were applied to mixture experiments and such models endure, where they are deemed appropriate. Claringbold (1955), Draper and Lawrence (1965a, 1965b), Thompson and Myers (1968), and Becker (1970) considered cases where the MIV are linear combinations of the component proportions, while Hackler, Kriegel, and Hader (1956) and Kenworthy (1963) took the MIV to be ratios of the component proportions. The utility of the Scheffé polynomials was extended by Draper and John (1977a, 1977b) and Chen, Zhu, and Hu (1985), who proposed the use of inverse terms and logarithmic terms, respectively, Gorman and Hinman (1962), who discussed a higher-order derivation, and Darroch and Waller (1985), Draper and Pukelsheim (1998), Cornell (2000), and Piepel, Szychowski, and Loeppky (2002), who each presented useful reparameterizations. An overview of mixture experiment methodology was given by Cornell (2002).

The terms of the existing mixture experiments models do not allow sufficient flexibility to accommodate differences in the way the components affect the response. The joint effects, that is, those described by terms involving two or more components, are limited. For example, existing models have limited capability to represent rapid change in the response in certain areas of the experimental region. As a result, models may represent the response surface inaccurately or with a greater number of terms than necessary.

Models that are nonlinear in parameters have not been applied to data from mixture experiments, with the exception of those given by Focke, Sandrock, and Kok (2007) and Focke, Ackermann, and Coetzer (2012). The models proposed by these authors have application for only a small number of components. However, there are situations where more complex models would be preferable over models providing potentially crude polynomial approximations. The models proposed by Becker (1968, 1978) provide some of the required increased flexibility. However, these models assume linear blending, or alternatively inactivity, of one or more components.

The class of models we propose include many of the Scheffé (1958, 1963) and Becker (1968, 1978) models as special cases. However, this class of models also encompasses other ideas for modeling mixture experiments.

In Section 2 we summarize the main features of mixture experiments. We also discuss different effects that mixture components may have. In Section 3 we introduce a new general class of mixture models and discuss its relation to existing models. We focus on models with binary and ternary blending as they are useful in practice. In Section 4 we show that choosing the appropriate model for a specific study and its estimation can be combined, thus leading to a simple model selection procedure that can be implemented using many statistical packages. This is demonstrated with a simulated example, chosen to illustrate a situation when the new models provide excellent fit of the data, while the standard models do not. The dataset and computer code implementing the analysis are available as supplementary material on the journal’s website. We conclude the article with a discussion of the advantages and limitations of the new methodology.

## 2. MIXTURE EXPERIMENTS

In mixture experiments the response of interest, *y*, is dependent on the proportions of the *q* mixture components *x_i_*, *i* = 1, …, *q*, such that

(1) $$\sum_{i=1}^{q}x_{i}=1,x_{i}\geq 0.$$

The unconstrained composition space of the experiment is the (*q* − 1)- dimensional simplex. However, individual component lower and upper bounds, linear multicomponent constraints, and nonlinear constraints (Atkinson, Donev, and Tobias 2007, p. 230) often apply.

Scheffé (1958) proposed the use of {*q*, *m*} symmetric canonical polynomial models obtained by reparameterization of standard polynomials of degree *m* for *q* components by using (1). The quadratic (S2), cubic (S3) and special cubic (SSC3) Scheffé polynomials for mixtures are

(2) $$\begin{array}{ccc} E[y] & = & \sum_{i=1}^{q}\beta_{i}x_{i}+\sum_{i\neq j}^{q}\beta_{ij}x_{i}x_{j},\\ E[y] & = & \sum_{i=1}^{q}\beta_{i}x_{i}+\sum_{i<j}^{q}\beta_{ij}x_{i}x_{j}\\ & & +\;\sum_{i<j}^{q}\gamma_{ij}x_{i}x_{j}\left(x_{i}-x_{j}\right)+\sum_{i<j<k}^{q}\beta_{ijk}x_{i}x_{j}x_{k}, \end{array}$$

and

$$E\left[y\right]=\sum_{i=1}^{q}\beta_{i}x_{i}+\sum_{i<j}^{q}\beta_{ij}x_{i}x_{j}+\sum_{i<j<k}^{q}\beta_{ijk}x_{i}x_{j}x_{k},$$

respectively, where β_1_, β_2_, …, γ_12_, γ_13_, … are the parameters that must be estimated using the data.

As an alternative to the quadratic Scheffé polynomial, Piepel, Szychowski, and Loeppky (2002) suggested partial quadratic mixture (PQM) models, which are reduced forms of the model

$$E\left[y\right]=\sum_{i=1}^{q}\beta_{i}x_{i}+\sum_{i<j}^{q}\beta_{ij}x_{i}x_{j}+\sum_{i=1}^{q}\beta_{ii}x_{i}^{2},$$

where up to *q*(*q* − 1)/2 terms of binary joint effects *x_i_x_j_* (*i* ≠ *j*) or square terms *x* ^2^_*i*_ are included in the model in addition to the linear terms.

A full PQM model provides a fit equivalent to the full quadratic Scheffé polynomial. However, a reduced PQM model, containing squared terms, may prove more parsimonious than reduced quadratic Scheffé polynomials (e.g., when one or more components have strong quadratic curvature effects). This may equivalently be said to be the case for the models proposed by Draper and Pukelsheim (1998).

Becker (1968) introduced models that allow for describing linear blending:

(3) $$\begin{array}{ccc} H1:E[y] & = & \sum_{i=1}^{q}\beta_{i}x_{i}+\sum_{i<j}^{q}\beta_{ij}\min\left(x_{i},x_{j}\right)\\ & & +\;\sum_{i<j<k}^{q}\beta_{ijk}\min\left(x_{i},x_{j},x_{k}\right)+\cdots ,\\ H2:E[y] & = & \sum_{i=1}^{q}\beta_{i}x_{i}+\sum_{i<j}^{q}\beta_{ij}\frac{x_{i}x_{j}}{x_{i}+x_{j}}\\ & & +\;\sum_{i<j<k}^{q}\beta_{ijk}\frac{x_{i}x_{j}x_{k}}{x_{i}+x_{j}+x_{k}}+\cdots , \end{array}$$

and

(4) $$\begin{array}{ccc} H3:E[y] & = & \sum_{i=1}^{q}\beta_{i}x_{i}+\sum_{i<j}^{q}\beta_{ij}\sqrt{x_{i}x_{j}}\\ & & +\;\sum_{i<j<k}^{q}\beta_{ijk}\sqrt{3x_{i}x_{j}x_{k}}+\cdots . \end{array}$$

Reports of applications of these models include those of Becker (1968), Snee (1973), Johnson and Zabik (1981), Chen, Li, and Jackson (1996), and Cornell (2002), among others, most of whom demonstrate them to be advantageously used in comparison to Scheffé polynomials.

Becker (1978) progressed to propose the general model form,

(5) $$\begin{array}{ccc} E[y] & = & \sum_{i=1}^{q}\beta_{i}x_{i}+\sum_{i<j}^{q}h\left(x_{i},x_{j}\right)\left(x_{i}+x_{j}\right)\\ & & +\;\sum_{i<j<k}^{q}h\left(x_{i},x_{j},x_{k}\right)\left(x_{i}+x_{j}+x_{k}\right)+\cdots \end{array}$$

of which the H2 and H3 models are each a special case, where *h*() are each homogenous of degree zero, that is, their effect remains consistent for all values *x_i_* + *x_j_*, where *x_i_* and *x_j_* remain in fixed relatively proportion, and similarly *x_i_* + *x_j_* + *x_k_*, where *x_i_*, *x_j_* and *x_k_* remain in fixed relative proportions. Reduced forms of this model form allow the linear blending effect of one (or more) components on the response to be described. This is useful when one or more components has an additive effect on the response, such as a dilutent. Becker suggested the nonlinear terms

$$h\left(x_{j},...,x_{k}\right)=\prod_{i<j}^{k}{\left(\frac{x_{i}}{x_{j}+\cdots +x_{k}}\right)}^{r_{i}}$$

which potentially provide greater flexibility in joint effects of the components. However, he gives little guidance on how these nonlinear parameters could be used, nor does he consider their estimation.

As described so far, the full statistical model chosen for a particular study may have too many terms describing joint effects, particularly if the number of components is large. Often only a subset of them will be needed. Stepwise regression (Efroymson 1965) can be applied to achieve this.

## 3. MODEL GENERALIZATION

The linear (in the parameters) models discussed thus far will describe well situations where their terms accommodate the specific joint effects of the mixture components. However, their terms do not accommodate particular nonadditive effects and this could lead them to perform poorly. They may not adequately represent the response or do so in a manner detrimental to model parsimony. This section proposes a general class of models which can represent responses of mixtures whose components have a wide range of different effects. We first discuss joint effects of two components and then extend the presented ideas to three components. The joint effects of more than three components are rarely considered when modeling mixture experiments using existing methodology and, therefore, are not considered here. We start by describing an idea for combining and generalizing standard binary blending models.

### 3.1 Motivation

The models

(6) $$E\left[y\right]=\sum_{i=1}^{q}\beta_{i}x_{i}+\beta_{ij}x_{i}x_{j},$$

and

(7) $$E\left[y\right]=\sum_{i=1}^{q}\beta_{i}x_{i}+\beta_{ij}\frac{x_{i}x_{j}}{x_{i}+x_{j}},$$

where 1 ⩽ *i*, *j* ⩽ *q*, *i* ≠ *j*, characterize the response surface in contrasting ways with respect to the joint effect of *x_i_* and *x_j_*. While (7) allows an additive blending effect through the linear blending of Becker’s (1968)) H2 model, (6) uses the quadratic blending effect of the Scheffé polynomial. This contrast can be seen along any ray where *x_i_* and *x_j_* remain in a fixed relative proportion. Models (6) and (7) differ by the form of their last term. Where more than one pair of mixture components demonstrate joint effects, the best model fit may be achieved where the blending effect of the term in (6) is used for one pair of components and that of the term in (7) for another (Johnson and Zabik 1981).

First, a generalized binary blending effect is defined by introducing the parameter *s_ij_* in the model

(8) $$E\left[y\right]=\sum_{i=1}^{q}\beta_{i}x_{i}+\beta_{ij}\left(\frac{x_{i}}{x_{i}+x_{j}}\right)\left(\frac{x_{j}}{x_{i}+x_{j}}\right){\left(x_{i}+x_{j}\right)}^{s_{ij}}.$$

The generalized binary blending term in (8) could be mathematically reduced to the form $x_{i}x_{j}{\left(x_{i}+x_{j}\right)}^{s_{ij}^{*}}$, but it is written that way to more easily see subsequently that the Scheffé and Becker H2 models are special cases.

**Figure 1:**
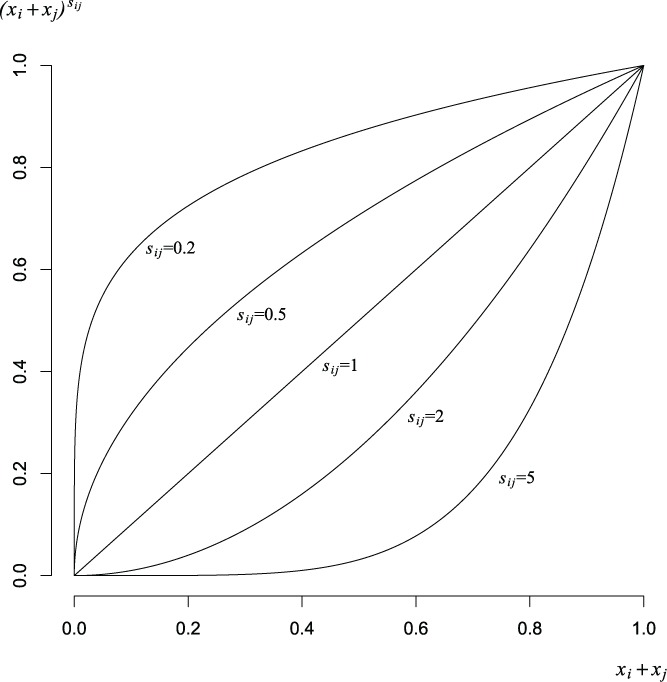
Blending effects for *s_ij_* = 0.2, 0.5, 1, 2, and 5.

The blending effects corresponding to five different values of *s_ij_* (*s_ij_* = 0.2, 0.5, 1, 2, 5) are shown in Figure 1. Note that increasing *s_ij_* above 1 results in a term whose effect is very small as *x_i_* + *x_j_* approaches zero, while reducing *s_ij_* toward 0 results in a term whose impact decreases rapidly as *x_i_* + *x_j_* approaches zero.

Further flexibility can be added by introducing *r_ij_* and *r_ji_* to the model, which gives

(9) $$E\left[y\right]=\sum_{i=1}^{q}\beta_{i}x_{i}+\beta_{ij}{\left(\frac{x_{i}}{x_{i}+x_{j}}\right)}^{r_{ij}}{\left(\frac{x_{j}}{x_{i}+x_{j}}\right)}^{r_{ji}}{\left(x_{i}+x_{j}\right)}^{s_{ij}},$$

where, if *s_ij_* = 1, this is a reduced form of (5) with only one term of joint effect. Model (9) is linear in the parameters β_*ij*_ for any values of the parameters *s_ij_*, *r_ij_*, *r_ji_* that define the form of the terms.

This concept can be extended to introduce a general ternary joint effect in the model:

(10) $$\begin{array}{ccc} E[y] & = & \sum_{i=1}^{q}\beta_{i}x_{i}+\beta_{ijk}{\left(\frac{x_{i}}{x_{i}+x_{j}+x_{k}}\right)}^{r_{ijk}}{\left(\frac{x_{j}}{x_{i}+x_{j}+x_{k}}\right)}^{r_{jki}}\\ & & \times \;{\left(\frac{x_{k}}{x_{i}+x_{j}+x_{k}}\right)}^{r_{kij}}{\left(x_{i}+x_{j}+x_{k}\right)}^{s_{ijk}}. \end{array}$$

Here, the joint effect of the *x_i_*, *x_j_*, and *x_k_* is governed by *s_ijk_*, *r_ijk_*, *r_jki_*, *r_kij_*, and the corresponding β_*ijk*_. In particular, *s_ijk_* governs the blending effect between *x_i_*, *x_j_*, and *x_k_* and the remainder of the mixture, in an analogous manner to *s_ij_* above. Thus, contrasting effects may be seen along any ray where *x_i_*, *x_j_*, and *x_k_* remain in fixed relative proportions. The new terms for the binary and ternary cases are referred to as generalized terms of binary and ternary joint effects.

Model (9) may alternatively be expressed as

$$\begin{array}{ccc} E[y] & = & \sum_{i=1}^{q}\beta_{i}x_{i}+\beta_{ij}{\left(\frac{x_{i}}{x_{i}+x_{j}}\right)}^{g_{ij}h_{ij}}\\ & & \times \;{\left(\frac{x_{j}}{x_{i}+x_{j}}\right)}^{g_{ji}\left(1-h_{ji}\right)}{\left(x_{i}+x_{j}\right)}^{s_{ij}}, \end{array}$$

where *g_ij_h_ij_* = *r_ij_* and *g_ij_*(1 − *h_ij_*) = *r_ji_*, so that *g_ij_* = *r_ij_* + *r_ji_* and *h_ij_* = *r_ij_*/*g_ij_*. This allows us to better interpret the effects *r_ij_* and *r_ji_* through constrained values of *h_ij_*, *g_ij_*, and *g_ji_*, where 0 ⩽ *h_ij_* ⩽ 1, *g_ij_* > 0 and *g_ji_* > 0.

**Figure 2:**
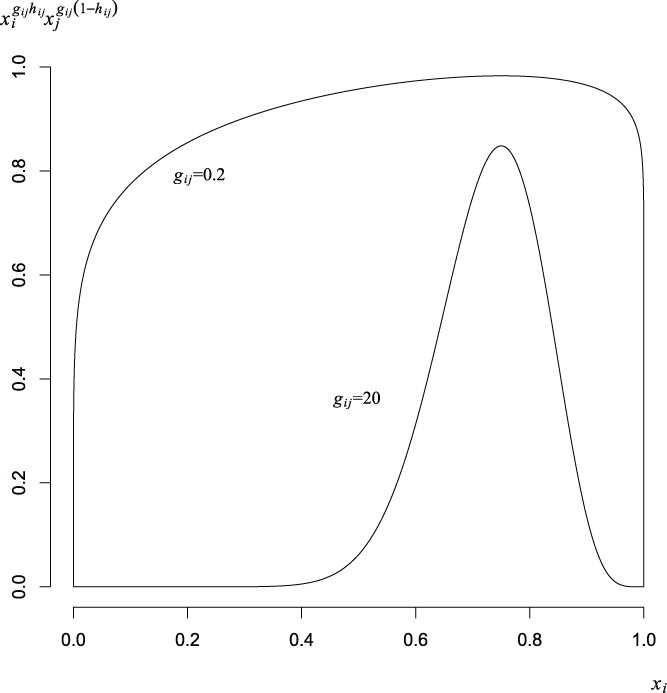
Binary blending effects for *g_ij_* = 0.2 and 20.

The interpretation of *g_ij_*, *g_ij_*, and *h_ij_* may be understood, without loss of generality, along the edge where *x_i_* + *x_j_* = 1. First *h_ij_* describes location, that is the point of greatest departure from linearity, with *h_ij_* = 0.5 indicating a symmetrical effect. Meanwhile, *g_ij_* defines the localization of the effect, where *g_ij_* ≫ 1 indicates a joint effect contained to a region localized about the point of greatest departure from linearity. In contrast, where *g_ij_* ≪ 1, the effect of the term changes little around this point but instead causes rapid change as *x_i_*/(*x_i_* + *x_j_*) approaches 0 and 1. The effect of the term is proportional to *x* ^*g_ij_h_ij_*^_*i*_ *x* ^*g_ij_*(1 − *h_ij_*)^_*j*_. To illustrate the way the shape changes with *x_i_* (or conversely with 1 − *x_j_*), the effects for *g_ij_* = 20 and *g_ij_* = 0.2 are shown in Figure 2 for *h_ij_* = 0.75.

A general binary term has no effect when *x_i_*/(*x_i_* + *x_j_*) or *x_j_*/(*x_i_* + *x_j_*) approaches zero. When *h_ij_* = 1 (or conversely, *h_ij_* = 0) the general binary blending term has greater significance as *x_i_*/(*x_i_* + *x_j_*) approaches 1, and *x_i_* dominates the effect. The manner in which this occurs is governed by *g_ij_*. For values of *g_ij_* > 1 the term becomes increasingly influential at an increasingly rapid rate as *x_i_*/(*x_i_* + *x_j_*) approaches 1. For values of *g_ij_* < 1 the term becomes increasingly influential at a decreasingly rapid rate. The impact of the term diminishes for larger values of *g_ij_*. The parameter β_*ij*_ sets the magnitude of the effect given its specification by *s_ij_*, *g_ij_*, and *h_ij_*.

Similar analysis may be made of the general term for a ternary joint effect. Model (10) may alternatively be expressed

$$\begin{array}{ccc} E[y] & = & \sum_{i=1}^{q}\beta_{ijk}x_{i}x_{j}x_{k}{\left(\frac{x_{i}}{x_{i}+x_{j}+x_{k}}\right)}^{g_{ijk}h_{ijk}}\\ & & \times \;{\left(\frac{x_{j}}{x_{i}+x_{j}+x_{k}}\right)}^{g_{ijk}h_{jki}}\\ & & \times \;{\left(\frac{x_{k}}{x_{i}+x_{j}+x_{k}}\right)}^{g_{ijk}\left(1-h_{ijk}-h_{jki}\right)}{\left(x_{i}+x_{j}+x_{k}\right)}^{s_{ijk}}, \end{array}$$

where *g_ijk_* = *r_ijk_* + *r_jki_* + *r_kij_*, *h_ijk_* = *r_ijk_*/*g_ijk_*, *h_jki_* = *r_jki_*/*g_ijk_*, *g_ijk_h_ijk_* = *r_ijk_*, *g_ijk_h_jki_* = *r_jki_*, and *g_ijk_*(1 − *h_ijk_* − *h_jki_*) = *r_kij_*. The new terms for the generalized ternary effects can be interpreted in a similar way as the generalized binary effects discussed earlier, without loss of the generality, as the effect of the term across the two-dimensional simplex where *x_i_* + *x_j_* + *x_k_* = 1. The parameters *h_ijk_* and *h_jki_* describe the location of the point of greatest departure from linearity, with *h_ijk_* = *h_jki_* = 1/3 indicating a rotationally symmetrical effect, while *g_ijk_* once again describes the localization of the effect about that point.

### 3.2 General Blending Mixture Models

We propose a class of generalized blending mixture (GBM) models, for *q* components, of the form

(11) $$\begin{array}{ccc} E[y] & = & \sum_{i=1}^{q}\beta_{i}x_{i}\\ & & +\;\sum_{i<j}\beta_{ij}{\left(\frac{x_{i}}{x_{i}+x_{j}}\right)}^{r_{ij}}{\left(\frac{x_{j}}{x_{i}+x_{j}}\right)}^{r_{ji}}{\left(x_{i}+x_{j}\right)}^{s_{ij}}\\ & & +\;\sum_{i<j<k}\beta_{ijk}{\left(\frac{x_{i}}{x_{i}+x_{j}+x_{k}}\right)}^{r_{ijk}}{\left(\frac{x_{j}}{x_{i}+x_{j}+x_{k}}\right)}^{r_{jki}}\\ & & \times \;{\left(\frac{x_{k}}{x_{i}+x_{j}+x_{k}}\right)}^{r_{kij}}{\left(x_{i}+x_{j}+x_{k}\right)}^{s_{ijk}}. \end{array}$$

In the second and third sums, we have q2 and q3 terms, respectively. Although one could have multiple terms involving the same variables with different powers, we do not consider this.

As discussed earlier, the GBM models can also be reparameterized as

(12) $$\begin{array}{ccc} & & E\left[y\right]=\sum_{i=1}^{q}\beta_{i}x_{i}\\ & & +\;\sum_{i<j}\beta_{ij}{\left(\frac{x_{i}}{x_{i}+x_{j}}\right)}^{g_{ij}h_{ij}}{\left(\frac{x_{j}}{x_{i}+x_{j}}\right)}^{g_{ji}\left(1-h_{ij}\right)}{\left(x_{i}+x_{j}\right)}^{s_{ij}}\\ & & +\;\sum_{i<j<k}\beta_{ijk}{\left(\frac{x_{i}}{x_{i}+x_{j}+x_{k}}\right)}^{g_{ijk}h_{ijk}}{\left(\frac{x_{j}}{x_{i}+x_{j}+x_{k}}\right)}^{g_{jki}h_{jki}}\\ & & \times \;{\left(\frac{x_{k}}{x_{i}+x_{j}+x_{k}}\right)}^{g_{kij}\left(1-h_{ijk}-h_{jki}\right)}{\left(x_{i}+x_{j}+x_{k}\right)}^{s_{ijk}}. \end{array}$$

Models (11) and (12) may be used to establish a broad range of joint effects. In fact, many models presented in the literature are special cases of our class of models. For example, the quadratic crossproduct terms in the Scheffé polynomial or the PQM model, occur when *h_ij_* = 0.5, *g_ij_* = 2, and *s_ij_* = 2. The squared terms in the PQM model occur when *h_ij_* = 0, *g_ij_* = 2, and *s_ij_* = 2. The binary blending terms of Becker’s H2 and H3 models occur when *h_ij_* = 0.5, *s_ij_* = 1, and *g_ij_* = 2 or 1, respectively. Furthermore, the ternary term of the special cubic model occurs when $h_{ijk}=h_{jim}=\frac{1}{3}$, *g_ijk_* = 3, and *s_ijk_* = 3. Thus, the GBM model allows us to consider commonly used terms, as well as new terms, with considerable flexibility.

## 4. MODEL SELECTION

Model (11) is complex, being a nonlinear function of some of its parameters. Its estimation is difficult but possible. However, in most cases it is unnecessary to estimate all its parameters simultaneously. When the parameters *r_ij_*, *r_ji_*, *s_ij_*, *r_ijk_*, *r_jik_*, and *s_ijk_* are specified, the estimation of the remaining parameters of (11) becomes trivial as the resulting models are linear in the parameters. Therefore, a sensible alternative to estimating (11) is to choose a model from a list of models that includes traditional models as well as new GBM models obtained for a grid of values for *r_ij_*, *r_ji_*, *s_ij_*, *r_ijk_*, *r_jik_*, and *s_ijk_*. The model selection criterion

$${\text{AIC}}_{c}=2pn/\left(n-p+1\right)-2\text{log}\left(L\right)$$

is used, where *L* is the likelihood function and *p* is the number of the parameters of the estimated model.

There are various ways of implementing such a comparison. The results presented here were obtained by a forward selection stepwise regression procedure, that is, by fitting first the model including the effects of the individual mixture components and then sequentially adding the best possible term representing joint action of two or three components, defined by all possible values of *r_ij_*, *r_ji_*, *s_ij_*, *r_ijk_*, *r_jik_*, and *s_ijk_* and judged by the AIC_*c*_ criterion. The fitting was terminated when the models became unnecessarily complicated. The computer implementation was done with the free software package R using the *AICc* function of the library *AICcmodavg* (Mazerolle 2013). The computer program is provided as online supplementary material.

Most published datasets obtained in mixture experiments are provided with satisfactory statistical analyses using standard models. Fitting GBM models to such data, therefore, usually brings modest benefits, which is not surprising. Furthermore, the experimental designs used in such studies often do not allow for fitting the GBM models, as they either have too few observations or their location in the design region does not allow the estimation of some of the model parameters. This is why to illustrate the features of the new models, data were simulated for a scenario where their advantages in comparison with standard models could be seen. Certainly, this is not a typical case and in most practical situations the differences seen in this example are likely to be considerably smaller.

**Figure 3:**
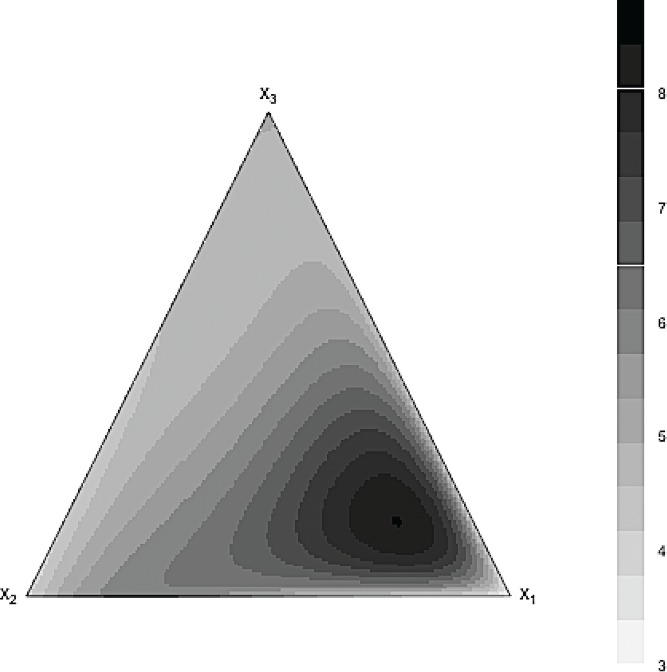
Contour plot for the underlying model.

*Example.* This example involves three mixture components with their proportions varying from 0 to 1. The response surface for this example was chosen to be asymmetric, but ordinary; see Figure 3. The maximum of the response was attained by a combination of a large proportion of *x*_1_ and similar but small proportions of the remaining components *x*_2_ and *x*_3_. However, the joint effect of the mixture components was strong. This was achieved by using the model

$$E\left[y\right]=3x_{1}+4x_{2}+5x_{3}+20x_{1}x_{2}^{3}+80\frac{x_{1}^{2.5}x_{2}^{0.5}x_{3}^{0.5}}{x_{1}+x_{2}+x_{3}}.$$

**Figure 4:**
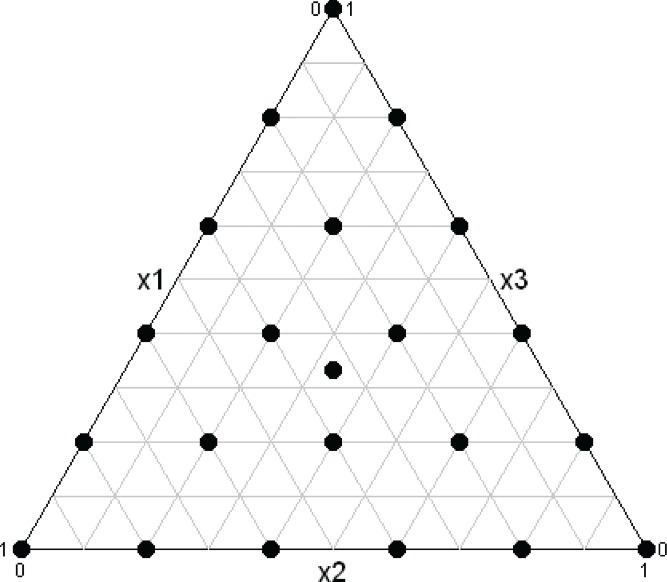
Plot of design for simulated data.

The data were generated for the 22-trial, 3-component simplex lattice design with an additional centroid point shown in Figure 4. Independent and normally distributed random errors with homogeneous variance equal to 0.25^2^ were added to the model-calculated values to yield the simulated data.

The best GBM model with four terms was chosen by comparing all possible models obtained by adding to the model having just three terms, that is,

$$E\left[y\right]=\sum_{i=1}^{3}\beta_{i}x_{i},$$

a single term of joint action. There were four types of terms to consider adding: $\beta_{ij}{\left(\frac{x_{i}}{x_{i}+x_{j}}\right)}^{r_{ij}}{\left(\frac{x_{j}}{x_{i}+x_{j}}\right)}^{r_{ji}}{\left(x_{i}+x_{j}\right)}^{s_{ij}}$, for *i* = 1, *j* = 2; *i* = 1, *j* = 3; and *i* = 2, *j* = 3; and β_123_ *x* ^*r*_123_^_1_ *x* ^*r*_231_^_2_ *x* ^*r*_312_^_3_, where the last term simplified from that in (11) as *x*_1_ + *x*_2_ + *x*_3_ = 1. Each of these terms was considered for all possible combinations of the values 0.5, 1, 1.5, 2, 2.5, or 3 for *r*_12_, *r*_13_, *r*_23_, *r*_123_, *r*_231,_ and *r*_312_ and the values 0, 1, 2, or 3 for *s*_12_, *s*_13_, and *s*_23_. Hence, 3(4*6^2^) + 6^3^ = 648 models were considered. The model with smallest AIC_*c*_ was chosen. It included a term representing binary blending for the components *x*_1_ and *x*_2_.

The best GBM model with five terms was chosen by comparing all models obtained by adding one more term to the best GBM model with four terms. The list of models to compare was obtained in the same way as that used to obtain the best GBM model with four terms. At this stage a term representing ternary blending for the components was included.

The same approach was used again to obtain the best GBM model with six and then, with seven terms. The terms that were added represented binary blending for components *x*_1_ and *x*_3_ and for components *x*_2_ and *x*_3_, respectively. As expected, the model with seven terms had the same structure as (11).

It may be beneficial to use different blending terms for the same components only in very rare situations. If this possibility is excluded in the model selection, it becomes faster as the number of models to consider becomes smaller as more terms are added to the model.

The full Scheffé cubic polynomial (2), Becker’s models H2 (3) and H3 (4) were also fitted to the data. Their AIC_*c*_ statistics, as well as those for the best GBM models with three, four, five, six, and seven terms, are given in Table 1.

**Table 1:** AIC_*c*_ statistics for models fitting the simulated data

Model	Scheffé	H2	H3	GBM	GBM	GBM	GBM	GBM
Terms	10	7	7	3	4	5	6	7
AIC_*c*_	95.85	80.53	78.72	80.60	45.53	9.91	10.49	11.81

The GBM model with five terms had a smaller AIC_*c*_ statistic than those for the GBM models with three, four, six, or seven terms, and was overall the best model. This model is

$$\begin{array}{ccc} \hat{y} & = & 2.8791\left(0.1366\right)x_{1}+3.8191\left(0.1585\right)x_{2}+5.1871\left(0.1279\right)x_{3}\\ & & +\;19.628\left(2.1260\right)x_{1}x_{2}^{2}+82.3909\left(3.6170\right)\frac{x_{1}^{2.5}x_{2}^{0.5}x_{3}^{0.5}}{x_{1}+x_{2}+x3}, \end{array}$$

where the figures in the brackets are the standard deviations of the estimates of the parameters that precede them. The AIC_*c*_ value for this model is 9.91 and compares rather favorably with the corresponding values for the fitted full Scheffé cubic polynomial and Becker’s H2 and H3 models for which it is 95.85, 80.53, and 78.72, respectively. While we emphasize that such advantageous differences in favor of the GBM models are not typical, this example shows that for certain studied phenomena they can be achieved.

**Figure 5:**
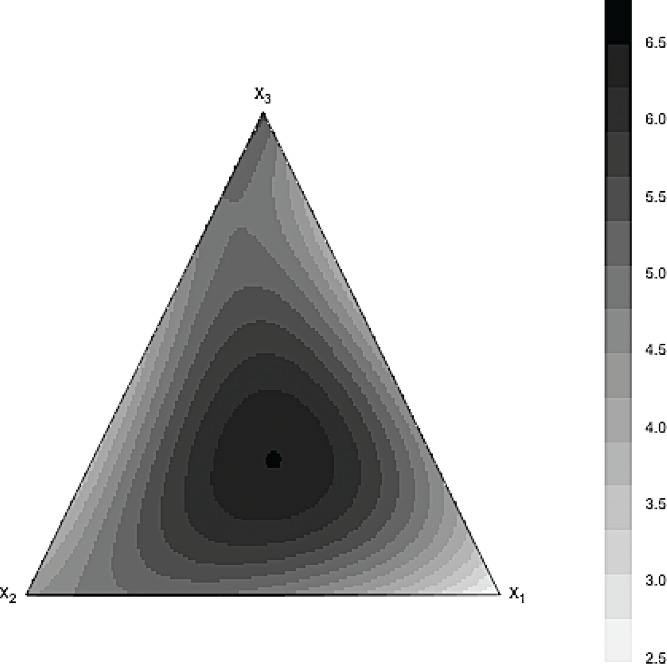
Prediction contour plot for Scheffé special cubic model fit to the simulated data.

**Figure 6:**
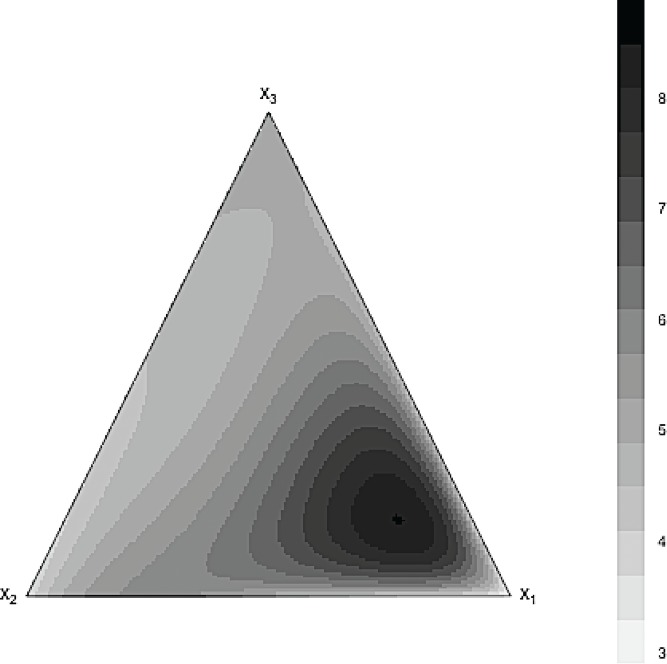
Prediction contour plot for GBM model fit to the simulated data.

The contour plots of the predicted surfaces with the full Scheffé cubic polynomial and the selected GBM model are shown in Figures 5 and 6. It can be seen that the estimated response surfaces of the GBM model was very similar to the true response surface shown in Figure 3. This cannot be said for the estimated full Scheffé cubic polynomial as its estimated response surface is notably different from the true surface.

Using a grid with a larger number of possible values for *r*_12_, *r*_13_, *r*_23_, *r*_123_, *r*_231_, *r*_312_, *s*_12_, *s*_13_, and *s*_23_ was attempted but did not bring any benefits. It was felt that the reason for that was that the amount of simulated data was not sufficiently large to allow to distinguish between models with such small differences of the values of the nonlinear parameters. However, as different models were found, some with structures somewhat different to that of the true model, they all produced predictions which would be considered indistinguishable in a practical application and well representing the underlying relationship.

## 5. DISCUSSION

The general class of models that we propose, provide a powerful unification and extension of the existing statistical methodology for analysis of data obtained in mixture experiments. The complexity of the models fitted to the data will closely match the complexity of the studied phenomena: they will be models equivalent to those proposed by Scheffé (1958, 1963), Becker (1968), and Piepel, Szychowski, and Loeppky (2002) when possible, but more complex when needed. The main benefits of using GBM models are that they are parsimonious and can accurately describe response surfaces in situations where sometimes standard models will offer only a crude and possibly even misleading approximation.

Estimating simultaneously all parameters of the GBM models (11) would require a substantial computational effort. The authors have made considerable progress in developing a computational tool capable of doing this, though its discussion remains outside the scope of the presented research. However, the method of choosing a GBM model proposed in this article is effective, simple, and computationally stable, thus it is good for its purpose.

The development of the general class of mixture models naturally creates the need to reevaluate the usefulness of the standard and computer generated experimental designs for mixture experiments. It is clear that fitting GBM models requires more data than fitting any of the standard models. It is possible to use space-filling experimental designs, collecting as much data as the available resources allow for. Such designs have been explored, for example, by Fang and Wang (1994), Borkowski and Piepel (2009), and Ning, Fang, and Zhou (2011). Further work aiming to formulate a better experimental design strategy for estimating the class of general blending mixture models that takes into account their structure is underway.

## SUPPLEMENTARY MATERIALS

**Data and Code:** The simulated dataset used in the example in Section 4 of the article, along with R code to perform the analysis (zip folder).

## Supplementary Material

Supplemental material
